# Supplementary figures and images for: An integrative monograph of Carex section Schoenoxiphium (Cyperaceae)
Source: PeerJ. 2021 May 17;9:e11336. doi: 10.7717/peerj.11336 (PMC8136282; doi:10.7717/peerj.11336)

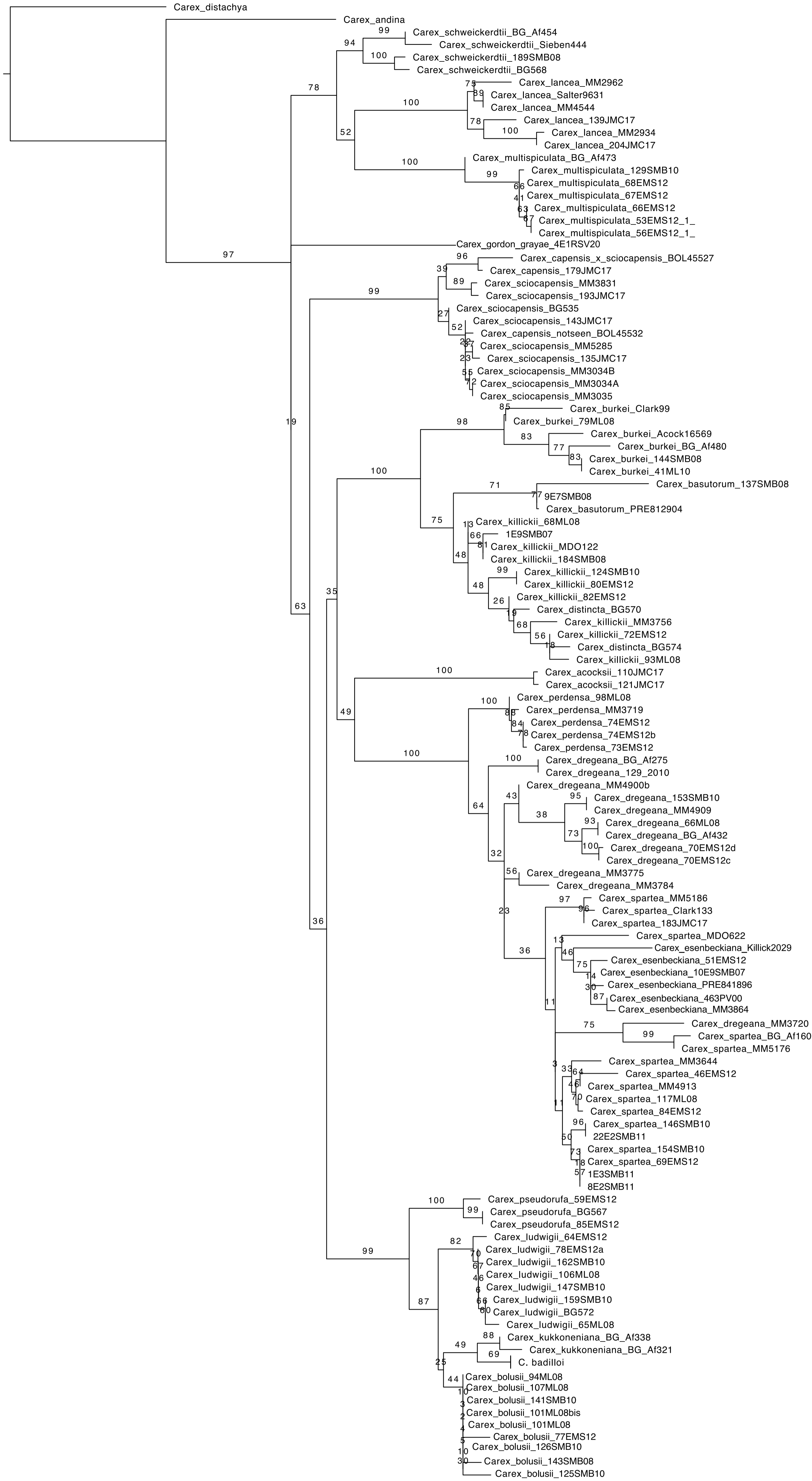

Supplement: Supplemental Information 5 — Maximum likelihood tree obtained from the RAxML analysis using a concatenated matrix of ETS, ITS, matK, and rps16 DNA regions (120 samples, 2,944 bp. Numbers above branches corresponding to bootstrap values are shown. [file peerj-09-11336-s005.pdf]

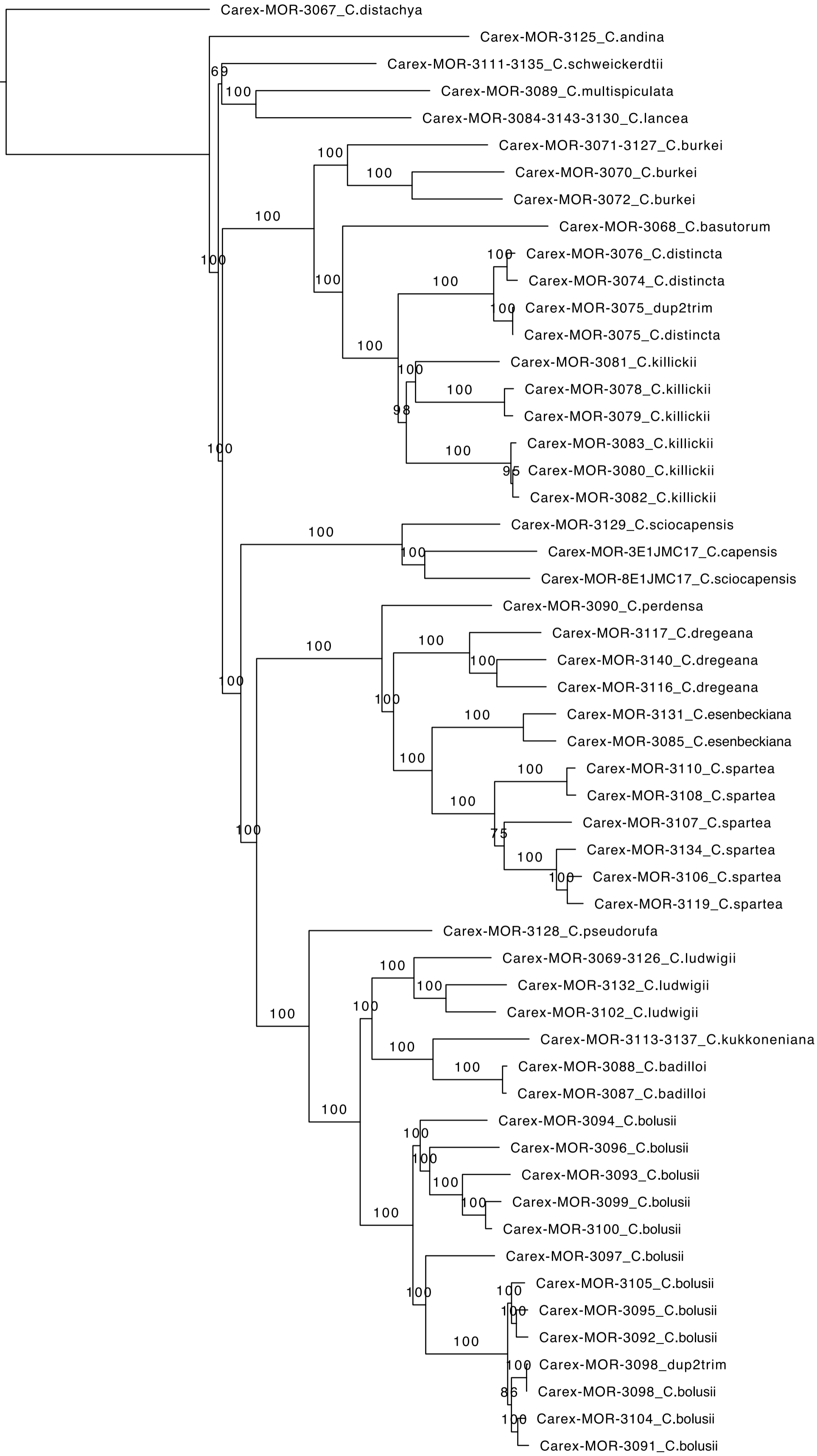

0.003

Supplement: Supplemental Information 6 — Maximum likelihood trees obtained from the RAxML analysis using a RAD-seq matrix of (54 samples, 4,478,844 bp). Numbers above branches corresponding to bootstrap values. [file peerj-09-11336-s006.pdf]
